# The effectiveness of sputum pH analysis in the prediction of response to therapy in patients with pulmonary tuberculosis

**DOI:** 10.7717/peerj.1448

**Published:** 2015-11-26

**Authors:** Makoto Masuda, Takashi Sato, Kentaro Sakamaki, Makoto Kudo, Takeshi Kaneko, Yoshiaki Ishigatsubo

**Affiliations:** 1 Department of Internal Medicine and Clinical Immunology, Yokohama City University Graduate School of Medicine, Yokohama, Japan; 2 Department of Pulmonology, Yokohama City University Graduate School of Medicine, Yokohama, Japan; 3 Department of Respiratory Medicine, Fujisawa City Hospital, Fujisawa, Japan; 4 Department of Biostatistics and Epidemiology, Yokohama City University Graduate School of Medicine, Yokohama, Japan

**Keywords:** Antituberculous therapy, Pulmonary tuberculosis, Airway acidity, Sputum pH

## Abstract

**Purpose.** The predictive factor of response to antituberculous therapy has not been fully elucidated. Airway acidity has been thought to be a potential indicator of the bactericidal activity. Therefore, we hypothesized that monitoring airway acidity by measuring sputum pH could predict response to therapy.

**Methods.** A total of 47 patients having newly diagnosed, smear-positive, active pulmonary tuberculosis were enrolled between October 2011 and March 2014. Sputum samples were serially analyzed before and after treatment. Eligible patients who initiated a standard 6-month treatment were monitored for the length of time to sputum smear and culture conversion.

**Results.** There were 39 patients who completed a 2-month intensive phase of isoniazid, rifampicin, pyrazinamide, and ethambutol therapy followed by a 4-month continuation phase of isoniazid and rifampicin. Although factors including age, cavitation, sputum grade, and use of an acid-suppressant were associated with initial low sputum pH in univariate analysis, multivariate analysis revealed that only age ≥61 years was a statistically important factor predicting low pH value (*p* = 0.005). Further outcome analysis showed that initial low sputum pH before treatment was the only factor significantly associated with shorter length of time to both sputum smear and culture conversion (*p* = 0.034 and 0.019, respectively) independent of the effects of age, sputum bacterial load, extent of lung lesion, and cavitation. Thus, initial low sputum pH indicated favorable response to anti-tuberculosis therapy.

**Conclusions.** Measuring sputum pH is an easy and inexpensive way of predicting response to standard combination therapy in patients with pulmonary tuberculosis.

## Introduction

Pulmonary tuberculosis (PTB) remains a major cause of death worldwide, accounting for 16.6% of newly diagnosed patients with PTB (an estimated 9.0 million) (World Health Organization, 2015a: Global Tuberculosis Report 2014) Antituberculous therapy using the combination of isoniazid (INH), rifampicin (RFP), ethambutol (EB), and pyrazinamide (PZA) has been established as an effective 6-month standard therapy, leading to a decrease in the global incidence of PTB (World Health Organization, 2010: Guidelines for treatment of tuberculosis). However, predictive factors of response to therapy have not been fully elucidated.

Among the standard therapy drugs, PZA has the potential to improve early bactericidal activity of other anti-tuberculosis combination drugs, such as INH-RFP or INH-streptomycin (Jindani et al., 1980). Hence, PZA has been considered a key drug, although the mechanism underlying this additive effect is unclear (Diacon et al., 2012; Gillespie et al., 2014; Jindani et al., 2014). In contrast, as PZA could raise concerns about liver dysfunction, especially when administered to elderly patients, the regimen without PZA would be initiated in clinical settings for safety reasons (Schaberg, Rebhan & Lode, 1996). However, patients treated without PZA receive a longer treatment period, which could raise other complications and poor adherence. Thus, the clarification of characteristics of patients who would be recommended for the PZA-including regimen would be important.

Based on the evidence showing that PZA had high bactericidal activity under acidic conditions (Salfinger & Heifets, 1988; Zhang et al., 1999), we hypothesized that monitoring airway pH could predict the response to therapy including PZA. The measurement of airway pH using exhaled breath condensate (EBC) has been reported to be useful for evaluating disease severity in patients with bronchial asthma and chronic obstructive pulmonary disease (COPD) (Antus et al., 2010; Kostikas et al., 2002; Papaioannou et al., 2011; Tseliou et al., 2010). Since collecting EBC in patients with PTB would raise safety concerns, we used fresh sputum samples. Our preliminary experiments demonstrated reliable and reproducible pH values in small amounts of sputum samples measured by a high-sensitive pH monitoring system.

This study examined the associations between sputum pH and length to sputum smear and/or culture conversion for the purpose of establishing markers predicting favorable outcomes in patients receiving standard 6-month antituberculous therapy.

## Materials & Methods

### Subjects

Patients enrolled in this study were newly diagnosed with sputum-microscopy-positive pulmonary tuberculosis and admitted to Yokohama City University Hospital between October 2011 and March 2014 for isolation and treatment. The prevalence of PTB patients in this area was 18 per 100,000 in 2011, which was the same as that in Japan (World Health Organization, 2015b: Tuberculosis country profiles). This study was approved by the Institutional Review Board (approval number: B110901018), and all patients provided written informed consent before study enrollment. Enrolled patients were initially treated with INH, RFP, PZA, and EB unless there was pre-existing liver disease or renal impairment. When a patient fulfilled the discharge criteria of at least 3 consecutive determinations of sputum-microscopy conversion (or alternatively at least 3 consecutive determinations of sputum culture conversion for patients showing continued expectoration of dead organisms), patients were referred to their local clinic to complete a standard course of therapy. The exclusion criteria of this study were as follows: subjects with malignancy, subjects who were pregnant, subjects who initiating treatment without PZA, and subjects who could not complete treatment due to intolerable side-effects.

### Sample collection

Sputum samples were serially obtained before and after initiating antituberculous therapy. Briefly, fresh sputum specimens were collected weekly, just after the patients woke-up, and kept under room temperature (around 22–24 °C) for further analysis. Samples containing bloody sputum were excluded from the data analysis due to possible unreliable pH measurements.

### Laboratory measurements

The sputum pH was electrically measured within 3 h of collection using a high-sensitive pH meter with the accuracy to the thousandth (0.001) (model F-71, Horiba, Japan). Medical records of each patient were reviewed. Sputum smears were confirmed by a standard fluorochrome procedure and bacterial load was graded based on the Japanese guidelines using the quantification scale (±, ≥1 acid-fast bacilli (AFB)/300 fields; 1 +, ≥1 AFB/100 fields; 2 +, ≥1 AFB/10 fields; 3 +, ≥10 AFB/fields) (Horita et al., 2012). Sputum for measurement of time to positivity of tuberculosis was assessed in a liquid culture medium (Middleback7H9). Culture before initiating treatment was tested for susceptibility to antituberculous drugs, and the minimum inhibitory concentrations of INH, RFP, EB and PZA as first-line drugs were examined. Chest radiographs were evaluated for the extent of lung parenchymal involvement and the presence of cavities. For monitoring toxicity, serum samples were serially collected, and other adverse effects, such as peripheral neuropathy and retro-bulbar optic neuropathy, were also evaluated.

### Data management and statistical analysis

We first evaluated possible independent factors affecting initial sputum pH, including age, sex, smoking history, sputum appearance, sputum bacterial load, use of an acid-suppressant, cavitation, and disease extent. These factors were analyzed by univariate and multivariate logistic regression analysis as dichotomous independent variables, using the following contrasts: age ≥61 vs. <61 (median); smoking history of current/ex-smoker vs. never smoker; sputum appearance of mucous vs. purulent; sputum bacterial load of ≥2+ vs. <2+; disease extent of ≥ one whole lung vs. <one lung; sputum pH ≥7.00 vs. <7.00 (median) (Sato et al., 2012). Next, we assessed outcome as defined by the time to smear and culture conversion in patients who completed a 2-month intensive phase of INH, RFP, PZA, and EB (HRZE) followed by a 4-month continuation phase of INH and RFP (HR). As reported previously, sputum bacterial load, extensive lung involvement, and presence of cavities have been demonstrated useful for predicting treatment outcome; therefore, we included these factors in addition to age and sputum pH (Fortun et al., 2007; Hesseling et al., 2010; Horne et al., 2010). Significant differences in sputum pH during storage or treatment were calculated using the paired *t*-test. Univariate analyses using chi-square test or Fisher’s exact test were used to compare across potential factors affecting sputum pH. Multivariate logistic regression analysis (forward) was performed to identify significant independent predictors. Independent variables were included in the model when the *p*-value was <0.20 in each variable because potential confounders should be eliminated only if *p* > 0.20 in order to prevent residual confounding (Horne et al., 2010; Maldonado & Greenland, 1993). The independence of factors affecting treatment outcome was evaluated by the Cox proportional hazards model. The time to sputum smear and culture conversion was assessed by the log-rank test. A two-tailed *p*-value of <0.05 was considered statistically significant. Continuous data were expressed as mean ± standard deviation (SD). Statistical analyzes were performed using MedCalc version 15 (Mariakerke, Belgium).

## Results

### Characteristics

Forty-seven patients were recruited and enrolled in this study. Of these, 1 patient with liver cancer and 1 pregnant patient were excluded. Furthermore, 2 patients who initiated treatment with HRE due to liver cirrhosis and 4 patients who discontinued treatment with HRZE (INH (5 mg/kg) + RFP (10 mg/kg) + (B [15 mg/kg) + PZA (25 mg/kg)) during the first 2-month intensive phase due to liver dysfunction (>5× normal value in an asymptomatic patient and 3× in a symptomatic patient) were excluded. The clinical characteristics of the remaining patients (*n* = 39) are summarized in Table 1. There were 25 male and 14 female patients, and their ages ranged from 16 to 87 years (median, 61 years). Smoking history and acid-suppressant therapy were considered potential factors affecting sputum pH, and thus were further analyzed. There were 17 current smokers and 14 ex-smokers (mean, 40 pack-years), therefore, our cohort might include COPD, although there was only one definitive COPD patient at the time of admission. Chest X-ray showed that 14 patients (35.9%) had extensive pulmonary lesions over one whole lung, and 24 patients (61.5%) had cavities. We confirmed that no patient had a history of bronchial asthma or was co-infected with HIV. Cultures from all patients showed favorable susceptibility to INH, RFP, EB, and PZA.

**Table 1:** Baseline characteristics of patients with pulmonary tuberculosis.

Characteristics	*N* = 39
Age, median year (range)	61 (16–87)
Gender—Male, No. (%)	25 (64.1)
Smoking history (current/ex-smoker/never)	17/14/8
Use of acid-suppressant, No. (%)^a^	14 (35.9)
Immunocompromised, No. (%) ^b^	13 (33.3)
Extensive lung lesion, No. (%)^c^	14 (35.9)
Presence of cavities, No. (%)	24 (61.5)
Sputum appearance, M1/M2/P1/P2/P3^d^	5/5/10/11/8
Sputum bacterial load, scanty /1 + /2 + /3 +	2/15/8/14
Sputum pH, median (range)	7.00 (5.50–8.37)

**Notes.**

a Acid-suppressant including histamine-2 receptor antagonist and proton pump inhibitor.

b Immunocompromised; Patients having diabetes mellitus and/or corticosteroid user.

c Extensive lung lesion; Radiological extent of parenchymal disease over one whole lung.

d Sputum appearance classified by Miller and Jones’ classification.

### Analysis of factors affecting sputum pH

**Figure 1:**
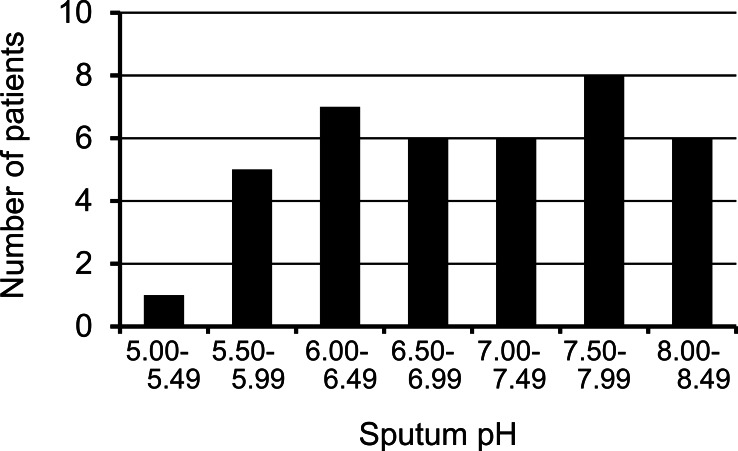
Distribution of initial sputum pH before treatment in pulmonary tuberculosis patients. The pH of freshly collected sputum samples was measured according to the Methods section. In the 39 patients included, the median value of initial sputum pH was 7.00.

**Figure 2:**
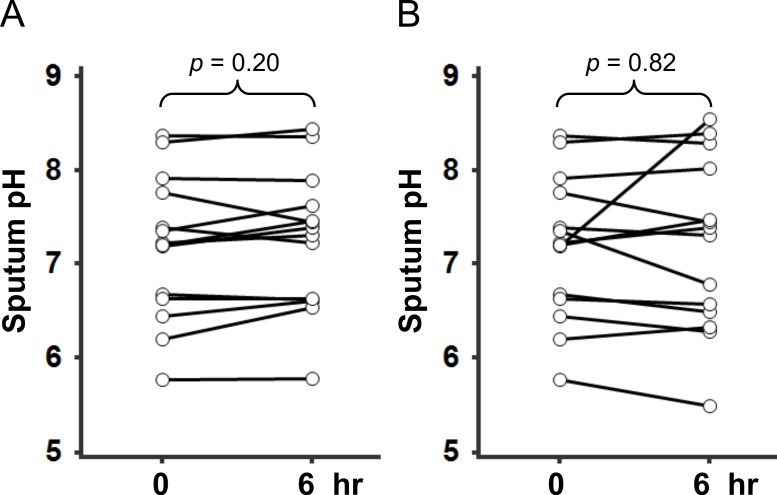
Changes in sputum pH after collecting samples in pulmonary tuberculosis patients. Serial analysis of pH in freshly collected sputum samples (*n* = 14) was made up to 6 h under deferent temperature conditions: (A) 4 °C or (B) room temperature. Statistical analysis was performed by using paired-*t* test.

**Figure 3:**
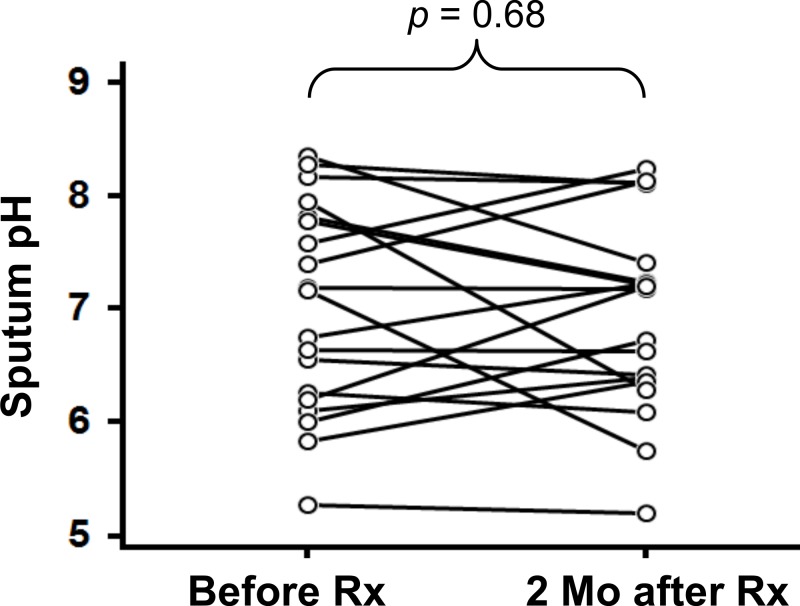
Changes in sputum pH before and after treatment in pulmonary tuberculosis patients. Paired sputum samples from patients before and 2 months after initiating antituberculous therapy are shown. Statistical analysis was paired *t*-test.

The sputum pH was uniformly-distributed between 5.50 and 8.37, with a median value of 7.00, from 39 included patients (Fig. 1 and Table 1). First, we examined whether the sputum pH could be reproducible under the conditions of (1) different time points after sample collection and (2) different temperatures of sample preservation. Our preliminary studies showed that the value of sputum pH did not differ up to 6 h after collection, regardless of storage temperature (Fig. 2). Of particular importance, there was no significant change in sputum pH before and 2 months after initiating treatment with HRZE (Fig. 3, *p* = 0.68, *n* = 19). The potential clinical characteristics associated with sputum pH were analyzed and summarized in Table 2. In univariate analysis, age ≥61 years was a statistically important factor predicting low pH value (*p* < 0.01 vs. aged <61 years). Of note, there was an inverse association between age and sputum pH value (*r* = − 0.56, *p* < 0.01, *n* = 39). Unexpectedly, smoking history and extensive lung lesions did not affect the sputum pH values. The use of an acid-suppressant (*p* = 0.048), being immunocompromised (*p* = 0.096), the presence of cavities (*p* = 0.105), and sputum bacterial load (*p* = 0.111) were potential predictors of sputum pH (Table 2). Multivariate analysis identified that only age ≥61 years was an independent predictor of low sputum pH (*p* = 0.005; odds ratio (OR) 24.535; 95% CI [2.685–224.213].

**Table 2:** Univariate and multivariate analyses of odds ratio for an initial low sputum pH (<7.00).

	Univariate analysis			Multivariate analysis		
Characteristics	OR	95% CI	*P* ^e^	OR	95% CI	*P* ^f^
Age ≥61 yr	8.750	2.100–36.251	0.004	24.535	2.685–224.213	0.005
Male sex	2.291	0.613–8.498	0.320			
Smoking history (current/ex-smoker vs. never)	0.938	0.214–4.108	1.000			
Use of acid-suppressant^a^	4.444	1.115–17.497	0.048			
Immunocompromised^b^	3.600	0.905–14.132	0.096			
Extensive lung lesion^c^	1.697	0.466–6.165	0.514			
Presence of cavities	0.300	0.080–1.130	0.105			
Sputum appearance^d^(M vs P)	0.542	0.134–2.219	0.480	0.104	0.010–1.128	0.063
Sputum bacterial load (<2+ vs ≥2+)	3.208	0.877–11.719	0.111			

**Notes.**

CI confidence interval OR odds ratio

a Acid-suppressant including histamine-2 receptor antagonist and proton pump inhibitor.

b Immunocompromised; Patients having diabetes mellitus and/or corticosteroid user.

c Extensive lung lesion; Radiological extent of parenchymal disease over one whole lung.

d Sputum appearance classified by Miller and Jones’ classification.

e Fisher’s exact test.

f ^¶^ Logistic regression.

### Analysis of sputum pH and outcome

**Figure 4:**
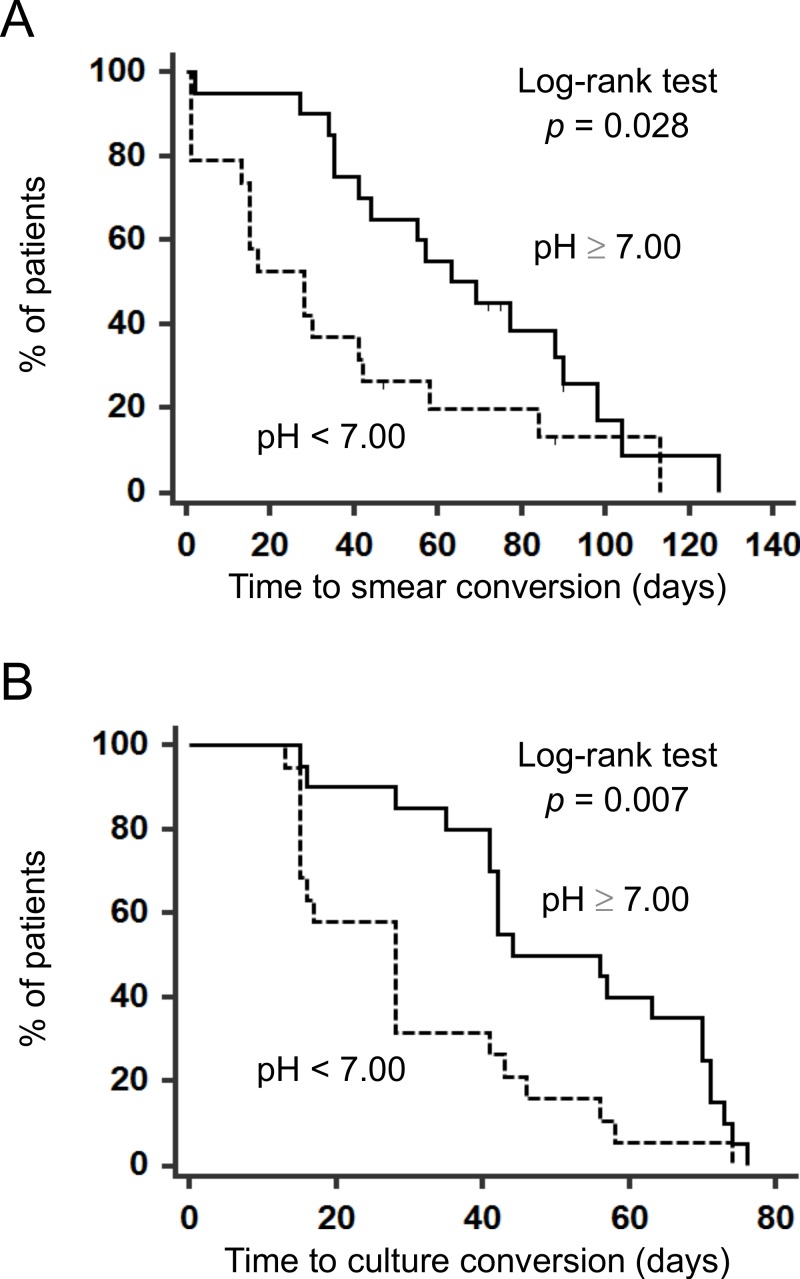
The time to sputum smear and culture conversion in pulmonary tuberculosis patients. Thirty-nine included patients were divided into groups according to median initial sputum pH. Kaplan–Meier Curves of time to (A) sputum smear conversion and (B) sputum culture conversion were made in low (pH < 7.00) and high (pH ≥ 7.00) sputum pH groups, and analyzed using the log-rank test. Ticks indicate censored data.

We further analyzed the association between initial sputum pH and clinically important outcome, including the time to, (1) smear conversion and (2) culture conversion of sputum, as useful indicators of response to treatment with HRZE. Among the 39 patients included, 5 patients showing persistent smear-positive results fulfilled the discharge criteria. Therefore, the remaining 34 patients were examined as evaluable smear-conversion subjects. There was a moderate positive relationship between initial sputum pH and time to smear conversion (*r* = 0.342, *p* = 0.048, *n* = 34). Similarly, initial sputum pH was modestly correlated with time to culture conversion (*r* = 0.304, *p* = 0.060, *n* = 39). Further, when patients were divided into initial sputum pH <7.00 vs. ≥7.00 (median for whole group) groups, the low pH group showed significantly shorter hospital stay as determined by time to smear conversion (29.6 ± 31.0 vs. 61.5 ± 32.5 days [mean ± SD], *p* = 0.028, log-rank test) or alternatively, time to culture conversion (30.5 ± 17.9 vs. 51.4 ± 19.4 days [mean ± SD], *p* = 0.007, log-rank test) as shown in Fig. 4. Since age was shown to be significantly correlated with sputum pH (Table 2), we evaluated the effect of age on outcome analysis. Also, known factors such as sputum bacterial load, extent of lung lesion, and cavity formation have been thought of as biomarkers to identify PTB patients at risk of longer hospital stays and relapse (Fortun et al., 2007; Hesseling et al., 2010; Horne et al., 2010), and thus be included in multivariate Cox regression analysis. Interestingly, although age ≥61 years is also a factor affecting initial low sputum pH, the lack of significance between age and response to therapy is identified (Tables 2 and 3). This is convincing because elder PTB patients exhibit higher mortality (Feng et al., 2011). Possible reasons of this dissociation are thought to be treatment interruption and/or discontinuation due to multiple organ dysfunctions caused by initiating therapy in elder patients. However, 14 (30%) of the 47 patients enrolled in the current study were aged 75 years or older, and the majority (*n* = 12, 86%) of these patients could continue HRZE, and could be discharged from hospital. Thus, older age may not always associate with higher mortality in PTB. Importantly, other potential factors such as sputum bacterial load, extensive lung involvement, and cavity formation did not affect the outcome analysis using a multivariate Cox regression model in our cohort (Table 3). Accordingly, sputum pH was found to be the most powerful independent predictor of the time to both sputum smear and culture conversion in patients receiving the standard 2-month HRZE followed by a 4-month HR therapy (Table 3, *p* = 0.034 and 0.019, respectively).

**Table 3:** Cox regression analysis for baseline predictors of sputum smear and culture conversion in patients.

	Predictor variable	Hazard ratio	95% CI	*P*
Sputum smear conversion (days)	Age ≥61 yr	1.910	0.724–5.036	0.193
	Sputum bacterial load^a^	1.465	0.644–3.334	0.366
	Extensive lung lesion ^b^	0.543	0.221–1.334	0.185
	Presence of cavities ^c^	1.825	0.845–3.941	0.128
	Sputum pH ≥7.0	3.094	1.093–8.760	0.034
Sputum culture conversion (days)	Age ≥61 yr	1.556	0.724–3.347	0.260
	Sputum bacterial load ^a^	1.390	0.650–2.974	0.398
	Extensive lung lesion^b^	0.723	0.322–1.623	0.434
	Presence of cavities ^c^	2.044	0.894–4.673	0.092
	Sputum pH ≥ 7.0	2.717	1.183–6.240	0.019

**Notes.**

CI confidence interval

a Dummy variables for sputum bacterial load: 0 for bacterial load <2+, 1 for bacterial load ≥2+.

b Dummy variables for extensive lung lesion: 0 for lung lesion < one whole lung, 1 for lung lesion ≥ one whole lung.

c Dummy variables for presence of cavities: 0 for no cavity, 1 for presence of ≥ one cavity.

## Discussion

The sputum sample provides important information in both infectious and non-infectious pulmonary diseases (Dimakou, Hillas & Bakakos, 2009; Kodric et al., 2007; Ugarte-Gil et al., 2013). Previous reports have documented that the analysis of pH in sputum or EBC could monitor the inflammatory status in various lung diseases, and might reflect the success of subsequent therapy (Antus et al., 2010; Hunt et al., 2000; Kostikas et al., 2002; Papaioannou et al., 2011). Although low pH in sputum or EBC indicates airway acidity, which disadvantageously affects host defense and immune activation (Sutto, Conner & Salathe, 2004; Trevani et al., 1999), several antibiotics, such as PZA, have more bactericidal activity in acidic conditions (Salfinger & Heifets, 1988; Zhang et al., 1999). Based on these facts, we hypothesized that measuring pH in sputum or EBC would be useful for predicting response to therapy in PTB, because they require long-term treatment with careful management of several side effects.

One serious concern was accidental exposure to medical staff, especially when collecting the EBC samples. In contrast, sputum samples can be easily collected and mandatory assessment of isolation in patients with smear-positive active PTB. In addition, measuring pH is quite easy using an electrode or even by dipstick test. Since there have been few reports on the analysis of sputum pH, we needed to establish an appropriate procedure of measurement and analysis.

Our preliminary experiment revealed that the pH value of sputum was uniformly distributed, and was reproducible up to 6 h under either cold conditions or room temperature (Figs. 1 and 2). Unfortunately, we could not establish the control value because collecting sputum samples from healthy subjects was quite difficult. However, EBC or induced sputum from healthy subjects revealed the normal pH value around 7.5–7.7. (Kodric et al., 2007; Kostikas et al., 2002; Vaughan et al., 2003). Our PTB patients showed a relatively acidic airway environment (pH = 7.02 ± 0.89). Low pH values of EBC and induced sputum were also addressed in patients with asthma, COPD, and acute lung injury, where such values were associated with resistance to therapy (Antus et al., 2010; Gessner et al., 2003; Papaioannou et al., 2011). However, current findings show, for the first time, that an initial low pH value of sputum in PTB patients was the most powerful indicator predicting a favorable response to standard combination therapy (Table 3 and Fig. 4).

Next, we considered the reason for good prognosis in PTB patients with low pH sputum. A potential benefit of low pH in the lung lesion is that PZA has much more bactericidal activity in acidic conditions (Salfinger & Heifets, 1988). When administered in an acidic lesion, PZA is easily degraded into an activated form of pyrazinoic acid and accumulated in bacterial cytoplasm, which facilitates bactericidal activity (Zhang et al., 1999). Second, bacteria, even tuberculosis, is thought to be intolerable to acidic conditions (Piddington, Kashkouli & Buchmeier, 2000). Acidic conditions and the administration of PZA could act synergistically to kill tuberculosis, and thus lead to favorable shorter hospital stays in PTB patients presenting with low sputum pH. Our findings could support the establishment of a future new shorter regimen including PZA and the selection of eligible patients for a PZA-including regimen.

The current study has several limitations. First, the number of patients is limited, and the patients were recruited from a single hospital, limiting the generalizability of treatment regimen and results. Actually, we have tried to apply intensified treatment with HRZE even in patients older than 80 years, and 91.4% (*n* = 43) of eligible patients initiated therapy with HRZE, and 90.7% (*n* = 39) of them successfully completed 2 months HRZE without serious adverse events. The remaining patients (17.0%, *n* = 8) could not initiate therapy including PZA, or discontinued PZA due to liver dysfunction. We could not compare the outcome with or without PZA, in this relatively small group. Second, our cohort consists of single ethnic Japanese patients with favorable susceptibility to INH, RFP, EB, and PZA. That is, relatively low prevalence of drug resistance PTB (approximately 3.2% to any drug including INH, RFP, and EB among newly diagnosed patients in Japan) should be considered (Tuberculosis Research Committee , 2015). Thus, our findings may not be applicable to other unique populations. Third, we could follow and collect samples until patients fulfilled the discharge criteria. Therefore, we could not determine if their sputum pH reversed to normal, around 7.7–8.0, after completing treatment. Since this should be clarified, we are planning to monitor long-term sputum pH with a large number of PTB patients as a validation cohort from another center.

## Conclusions

Airway acidity is easily monitored using sputum samples, which might enable us to predict response to standard intensified therapy of HRZE in patients with PTB.

## Supplemental Information

10.7717/peerj.1448/supp-1 Data S1 Raw dataset of 39 included patients

10.7717/peerj.1448/supp-2 Data S2 Raw dataset of 39 included patients for logistic analysis

## Additional Information and Declarations

### Data Availability

The following information was supplied regarding data availability: The summarized dataset sheets are provided as Datas S1 and S2.

## References

[ref-1] Antus B, Barta I, Kullmann T, Lazar Z, Valyon M, Horvath I, Csiszer E (2010). Assessment of exhaled breath condensate pH in exacerbations of asthma and chronic obstructive pulmonary disease: a longitudinal study. American Journal of Respiratory and Critical Care Medicine.

[ref-2] Diacon AH, Dawson R, Von Groote-Bidlingmaier F, Symons G, Venter A, Donald PR, Van Niekerk C, Everitt D, Winter H, Becker P, Mendel CM, Spigelman MK (2012). 14-day bactericidal activity of PA-824, bedaquiline, pyrazinamide, and moxifloxacin combinations: a randomised trial. Lancet.

[ref-3] Dimakou K, Hillas G, Bakakos P (2009). Adenosine deaminase activity and its isoenzymes in the sputum of patients with pulmonary tuberculosis. International Journal of Tuberculosis and Lung Disease.

[ref-4] Feng JY, Su WJ, Chiu YC, Huang SF, Lin YY, Huang RM, Lin CH, Hwang JJ, Lee JJ, Yu MC, Yu KW, Lee YC (2011). Initial presentations predict mortality in pulmonary tuberculosis patients—a prospective observational study. PLoS ONE.

[ref-5] Fortun J, Martin-Davila P, Molina A, Navas E, Hermida JM, Cobo J, Gomez-Mampaso E, Moreno S (2007). Sputum conversion among patients with pulmonary tuberculosis: are there implications for removal of respiratory isolation?. Journal of Antimicrobial Chemotherapy.

[ref-6] Gessner C, Hammerschmidt S, Kuhn H, Seyfarth HJ, Sack U, Engelmann L, Schauer J, Wirtz H (2003). Exhaled breath condensate acidification in acute lung injury. Respiratory Medicine.

[ref-7] Gillespie SH, Crook AM, McHugh TD, Mendel CM, Meredith SK, Murray SR, Pappas F, Phillips PP, Nunn AJ, Consortium RE (2014). Four-month moxifloxacin-based regimens for drug-sensitive tuberculosis. New England Journal of Medicine.

[ref-8] Hesseling AC, Walzl G, Enarson DA, Carroll NM, Duncan K, Lukey PT, Lombard C, Donald PR, Lawrence KA, Gie RP, van Helden PD, Beyers N (2010). Baseline sputum time to detection predicts month two culture conversion and relapse in non-HIV-infected patients. International Journal of Tuberculosis and Lung Disease.

[ref-9] Horita N, Miyazawa N, Yoshiyama T, Kojima R, Omori N, Inoue M, Kaneko T, Ishigatsubo Y (2012). The presence of pretreatment cavitations and the bacterial load on smears predict tuberculosis infectivity negative conversion judged on sputum smear or culture. Internal Medicine.

[ref-10] Horne DJ, Johnson CO, Oren E, Spitters C, Narita M (2010). How soon should patients with smear-positive tuberculosis be released from inpatient isolation?. Infection Control and Hospital Epidemiology.

[ref-11] Hunt JF, Fang K, Malik R, Snyder A, Malhotra N, Platts-Mills TA, Gaston B (2000). Endogenous airway acidification. Implications for asthma pathophysiology. American Journal of Respiratory and Critical Care Medicine.

[ref-12] Jindani A, Aber VR, Edwards EA, Mitchison DA (1980). The early bactericidal activity of drugs in patients with pulmonary tuberculosis. American Review of Respiratory Disease.

[ref-13] Jindani A, Harrison TS, Nunn AJ, Phillips PP, Churchyard GJ, Charalambous S, Hatherill M, Geldenhuys H, McIlleron HM, Zvada SP, Mungofa S, Shah NA, Zizhou S, Magweta L, Shepherd J, Nyirenda S, van Dijk JH, Clouting HE, Coleman D, Bateson AL, McHugh TD, Butcher PD, Mitchison DA, Team RT (2014). High-dose rifapentine with moxifloxacin for pulmonary tuberculosis. New England Journal of Medicine.

[ref-14] Kodric M, Shah AN, Fabbri LM, Confalonieri M (2007). An investigation of airway acidification in asthma using induced sputum: a study of feasibility and correlation. American Journal of Respiratory and Critical Care Medicine.

[ref-15] Kostikas K, Papatheodorou G, Ganas K, Psathakis K, Panagou P, Loukides S (2002). pH in expired breath condensate of patients with inflammatory airway diseases. American Journal of Respiratory and Critical Care Medicine.

[ref-16] Maldonado G, Greenland S (1993). Simulation study of confounder-selection strategies. American Journal of Epidemiology.

[ref-17] Papaioannou AI, Loukides S, Minas M, Kontogianni K, Bakakos P, Gourgoulianis KI, Alchanatis M, Papiris S, Kostikas K (2011). Exhaled breath condensate pH as a biomarker of COPD severity in ex-smokers. Respiratory Research.

[ref-18] Piddington DL, Kashkouli A, Buchmeier NA (2000). Growth of Mycobacterium tuberculosis in a defined medium is very restricted by acid pH and Mg(2+) levels. Infection and Immunity.

[ref-19] Salfinger M, Heifets LB (1988). Determination of pyrazinamide MICs for Mycobacterium tuberculosis at different pHs by the radiometric method. Antimicrobial Agents and Chemotherapy.

[ref-20] Sato T, Saito Y, Inoue S, Shimosato T, Takagi S, Kaneko T, Ishigatsubo Y (2012). Serum heme oxygenase-1 as a marker of lung function decline in patients with chronic silicosis. Journal of Occupational and Environmental Medicine.

[ref-21] Schaberg T, Rebhan K, Lode H (1996). Risk factors for side-effects of isoniazid, rifampin and pyrazinamide in patients hospitalized for pulmonary tuberculosis. European Respiratory Journal.

[ref-22] Sutto Z, Conner GE, Salathe M (2004). Regulation of human airway ciliary beat frequency by intracellular pH. Journal of Physiology.

[ref-23] Trevani AS, Andonegui G, Giordano M, Lopez DH, Gamberale R, Minucci F, Geffner JR (1999). Extracellular acidification induces human neutrophil activation. Journal of Immunology.

[ref-24] Tseliou E, Bessa V, Hillas G, Delimpoura V, Papadaki G, Roussos C, Papiris S, Bakakos P, Loukides S (2010). Exhaled nitric oxide and exhaled breath condensate pH in severe refractory asthma. Chest.

[ref-25] Tuberculosis Research Committee TJ (2015). Nationwide survey of anti-tuberculosis drug resistance in Japan. International Journal of Tuberculosis and Lung Disease.

[ref-26] Ugarte-Gil CA, Elkington P, Gilman RH, Coronel J, Tezera LB, Bernabe-Ortiz A, Gotuzzo E, Friedland JS, Moore DA (2013). Induced sputum MMP-1, -3 & -8 concentrations during treatment of tuberculosis. PLoS ONE.

[ref-27] Vaughan J, Ngamtrakulpanit L, Pajewski TN, Turner R, Nguyen TA, Smith A, Urban P, Hom S, Gaston B, Hunt J (2003). Exhaled breath condensate pH is a robust and reproducible assay of airway acidity. European Respiratory Journal.

[ref-28] World Health Organization (2010). Guidelines for treatment of tuberculosis.

[ref-29] World Health Organization (2015a). Global Tuberculosis Report 2014.

[ref-30] World Health Organization (2015b). Tuberculosis country profiles.

[ref-31] Zhang Y, Scorpio A, Nikaido H, Sun Z (1999). Role of acid pH and deficient efflux of pyrazinoic acid in unique susceptibility of Mycobacterium tuberculosis to pyrazinamide. Journal of Bacteriology.

