# Supplementary material for: The effectiveness of sputum pH analysis in the prediction of response to therapy in patients with pulmonary tuberculosis
Source: PeerJ. 2015 Nov 26;3:e1448. doi: 10.7717/peerj.1448 (PMC4671190; doi:10.7717/peerj.1448)
Supplement: Data S1 [file peerj-03-1448-s001.pdf]

Gr	Patient No.	M/F =1/2	Age	Past history	Factors of compromised	Using PPI/H2	Pattern of disease area	Cavitation	extensive	pl	Date of testing (YYYY/MM/DD)	Date of smear conversion (YYYY/MM/DD)	Days of smear conversion (* = censored)	Date of culture conversion (YYYY/MM/DD)	Days of culture conversion	Sputum appearance Miller and Jones' classification	Sputum smear grade	Sputum pH	Temperature
pH<7.000	33	2	79	DM	DM	H2-blocker	b	Y	W		20110603	N/A	*47	20110719	46	P3	3+	5.496	25.0
	44	1	66	Alcoholism		–	b	N	W		20121127	20130307	113	20121212	28	P	2+	5.535	23.6
	30	1	87	COPD		PPI	b	Y	W		20110623	20110708	15	20110708	15	M2	2+	5.719	25.9
	15	1	67	DM	DM	–	r	Y	N		20111213	20111214	1	20111226	13	P2	±	5.829	24.8
	28	1	70	DM	DM	–	b	N	W		20131217	20140114	28	20140114	28	P1	3+	5.87	21.0
	57	2	55	Thrombocytopenia	PSL	PPI	b	N	W		20120329	20120413	15	20120413	15	P1	2+	5.999	23.7
	17	2	59	SLE	PSL	H2-blocker	b	N	N		20111024	20111025	1	20111108	15	M1	1+	6.002	23.3
	21	1	82			–	b	N	N		20111205	20111206	1	20111220	15	M1	1+	6.041	23.3
	19	1	63	DM	DM	–	b	Y	W	pl	20110324	N/A	*88	20110607	74	P3	3+	6.097	23.0
	7	1	86			H2-blocker	b	Y	W		20110912	20111012	30	20111107	56	P2	1+	6.106	24.2
	50	2	61			–	b	N	N		20120206	20120207	1	20120305	28	P2	1+	6.198	25.1
	9	1	85	PMR	PSL	PPI	b	N	N		20130422	20130619	58	20130619	58	P3	3+	6.423	24.2
	45	1	66	Schizopheenia		–	b	Y	N		20120116	20120409	84	20120228	43	P1	1+	6.491	24.0
	29	1	77			–	l	Y	N		20120326	20120410	15	20120410	15	P2	1+	6.568	23.9
	51	1	67			PPI	l	N	N		20120104	20120201	28	20120201	28	P	1+	6.635	24.8
	46	1	49	HCV		PPI	b	Y	W	pl	20111110	20111221	41	20111221	41	M1	1+	6.804	21.5
	35	1	79	Interstitial pneumonia	PSL	PPI	b	Y	N	pl	20130604	20130618	17	20130618	17	M2	3+	6.824	24.3
	12	2	79	Encepharitis	PSL	H2-blocker	l	N	N		20110704	20110815	42	20110720	16	P2	1+	6.878	24.0
	2	1	26			–	b	N	N		20130125	20130207	13	20130222	28	M1	1+	6.886	25.2
	N	19	19								19	17	17	19	19	0		19	19
	Mean	M/F=14/5	68.58				b=bilateral	Y=yes	W=over hemilung				29.6		30.5	M1/2= 4/2		6.232	23.9
	SD		15.04				r=right only	N=no	N=within hemilung				31.0		17.9	P1/2/3=5/5/3		0.451	1.2
	SEM		3.45				l=left only						7.5		4.1			0.104	0.3
pH>=7.000	1	2	33			–	b	N	N		20140129	20140402	63	20140312	42	P2	1+	7.008	22.5
	16	1	64	DM	DM	–	b	Y	N		20140214	20140320	34	20140327	41	P3	3+	7.102	22.0
	23	1	61	DM	DM	PPI	b	Y	N		20110804	N/A	*75	20111017	74	P1	1+	7.186	23.0
	26	1	64			–	b	Y	W		20110909	20111206	88	20111121	73	P1	2+	7.201	24.1
	52	1	51			–	b	Y	W		20130408	N/A	*72	20130618	71	P3	2+	7.244	23.8
	47	2	29			–	r	Y	N		20110630	20111006	98	20110825	56	P3	1+	7.483	24.0
	27	1	40			–	b	Y	W		20111006	20120104	90	20111216	71	P3	3+	7.577	25.7
	32	1	58			–	l	Y	N		20120327	20120801	127	20120605	70	P2	3+	7.812	23.3
	11	2	31			–	b	Y	N		20131125	20131230	35	20131230	35	M2	3+	7.852	25.5
	49	1	50	DM	DM	–	r	Y	N		20121024	20130205	104	20130108	76	P2	3+	7.858	23.8
	54	1	53			H2-blocker	r	Y	N		20111205	20120118	44	20120118	44	P1	3+	7.921	23.3
	38	2	79	MG, DM	DM	PPI	r	N	N		20110930	20111124	55	20111110	41	P2	1+	7.951	22.5
	14	2	16			–	b	N	N	pl	20140109	20140213	35	20140220	42	M2	1+	7.956	24.0
	4	1	18			–	l	N	N		20110920	20110922	2	20110922	16	M2	2+	7.996	24.0
	42	2	39			–	b	Y	W		20111214	20120229	77	20120215	63	P2	3+	8.011	23.4
	24	2	28			–	b	Y	N		20110630	20110810	41	20110811	42	P1	±	8.115	24.3
	5	2	64			–	b	Y	W		20111007	N/A	*90	20111216	70	P2	3+	8.164	23.7
	22	2	83			–	b	N	W		20130305	20130501	57	20130501	57	P3	2+	8.223	24.5
	6	1	40			–	l	Y	N		20110817	20110913	27	20110914	28	P1	3+	8.356	26.7
	43	1	53	Alcoholism		H2-blocker	b	Y	N		20130307	20130510	69	20130322	15	M1	2+	8.367	24.6
	N	20	20								20	17	17	20	20	0	0	20	20
	Mean	M = 11	47.70			P/H=2/2	b=bilateral	Y=yes	W=over hemilung				61.5		51.4	M1/2=1/3		7.769	23.9
	SD	F = 9	18.77				r=right only	N=no	N=within hemilung				32.5		19.4	P1/2/3=5/6/5		0.428	1.1
	SEM		4.20				l=left only						7.9		4.3			0.096	0.3
