# Supplementary material for: The effectiveness of sputum pH analysis in the prediction of response to therapy in patients with pulmonary tuberculosis
Source: PeerJ. 2015 Nov 26;3:e1448. doi: 10.7717/peerj.1448 (PMC4671190; doi:10.7717/peerj.1448)
Supplement: Data S2 [file peerj-03-1448-s002.pdf]

| Gr | Patient No. | Age (1>60y.o.) | Immunocompromized (1=yes) | PPI/H2 (1=user) | Cavities (1=yes) | Extensive lung lesion (1=>one lung) | Sputum bacterial load (<2+ vs =>2+) |
|---|---|---|---|---|---|---|---|
| pH<7.000 (=1) | 33 | 1 | 1 | 1 | 1 | 1 | 1 |
| | 44 | 1 | 0 | 0 | 0 | 1 | 1 |
| | 30 | 1 | 0 | 1 | 1 | 1 | 1 |
| | 15 | 1 | 1 | 0 | 1 | 0 | 0 |
| | 28 | 1 | 1 | 0 | 0 | 1 | 1 |
| | 57 | 0 | 1 | 1 | 0 | 1 | 1 |
| | 17 | 0 | 1 | 1 | 0 | 0 | 0 |
| | 21 | 1 | 0 | 0 | 0 | 0 | 0 |
| | 19 | 1 | 1 | 0 | 1 | 1 | 1 |
| | 7 | 1 | 0 | 1 | 1 | 1 | 0 |
| | 50 | 1 | 0 | 0 | 0 | 0 | 0 |
| | 9 | 1 | 1 | 1 | 0 | 0 | 1 |
| | 45 | 1 | 0 | 0 | 1 | 0 | 0 |
| | 29 | 1 | 0 | 0 | 1 | 0 | 0 |
| | 51 | 1 | 0 | 1 | 0 | 0 | 0 |
| | 46 | 0 | 0 | 1 | 1 | 1 | 0 |
| | 35 | 1 | 1 | 1 | 1 | 0 | 1 |
| | 12 | 1 | 1 | 1 | 0 | 0 | 0 |
| | 2 | 0 | 0 | 0 | 0 | 0 | 0 |
| | N | 19 | 19 | 19 | 19 | 19 | 19 |
| | Mean | | | | | | |
| | SD | | | | | | |
| | SEM | | | | | | |
| pH>=7.000 (=0) | 1 | 0 | 0 | 0 | 0 | 0 | 0 |
| | 16 | 1 | 1 | 0 | 1 | 0 | 1 |
| | 23 | 1 | 1 | 1 | 1 | 0 | 0 |
| | 26 | 1 | 0 | 0 | 1 | 1 | 1 |
| | 52 | 0 | 0 | 0 | 1 | 1 | 1 |
| | 47 | 0 | 0 | 0 | 1 | 0 | 0 |
| | 27 | 0 | 0 | 0 | 1 | 1 | 1 |
| | 32 | 0 | 0 | 0 | 1 | 0 | 1 |
| | 11 | 0 | 0 | 0 | 1 | 0 | 1 |
| | 49 | 0 | 1 | 0 | 1 | 0 | 1 |
| | 54 | 0 | 0 | 1 | 1 | 0 | 1 |
| | 38 | 1 | 1 | 1 | 0 | 0 | 0 |
| | 14 | 0 | 0 | 0 | 0 | 0 | 0 |
| | 4 | 0 | 0 | 0 | 0 | 0 | 1 |
| | 42 | 0 | 0 | 0 | 1 | 1 | 1 |
| | 24 | 0 | 0 | 0 | 1 | 0 | 0 |
| | 5 | 1 | 0 | 0 | 1 | 1 | 1 |
| | 22 | 1 | 0 | 0 | 0 | 1 | 1 |
| | 6 | 0 | 0 | 0 | 1 | 0 | 1 |
| | 43 | 0 | 0 | 1 | 1 | 0 | 1 |
| | N | 20 | 20 | 20 | 20 | 20 | 20 |
| | Mean | | | | | | |
| | SD | | | | | | |
| | SEM | | | | | | |
